# Perceptions about the Federally Mandated Smoke-Free Housing Policy among Residents Living in Public Housing in New York City

**DOI:** 10.3390/ijerph15102062

**Published:** 2018-09-20

**Authors:** Nan Jiang, Lorna Thorpe, Sue Kaplan, Donna Shelley

**Affiliations:** Department of Population Health, School of Medicine, New York University, New York, NY 10016, USA; nan.jiang2@nyulangone.org (N.J.); lorna.thorpe@nyulangone.org (L.T.); sue.kaplan@nyulangone.org (S.K.)

**Keywords:** public housing, multiunit housing, smoke-free law

## Abstract

*Background*: To assess residents’ attitudes towards the United States (U.S.) Department of Housing and Urban Development’s new smoke-free public housing policy, perceptions about barriers to policy implementation, and suggestions for optimizing implementation. *Methods*: In 2017, we conducted 10 focus groups among 91 residents (smokers and nonsmokers) living in New York City public housing. *Results*: Smokers and nonsmokers expressed skepticism about the public housing authority’s capacity to enforce the policy due to widespread violations of the current smoke-free policy in common areas and pervasive use of marijuana in buildings. Most believed that resident engagement in the roll-out and providing smoking cessation services was important for compliance. Resident expressed concerns about evictions and worried that other building priorities (i.e., repairs, drug use) would be ignored with the focus now on smoke-free housing. *Conclusions*: Resident-endorsed strategies to optimize implementation effectiveness include improving the access to cessation services, ongoing resident engagement, education and communication to address misconceptions and concerns about enforcement, and placing smoke-free homes in a larger public housing authority healthy housing agenda.

## 1. Introduction

On 30 July 2018, the United States (U.S.) Department of Housing and Urban Development’s (HUD) mandate on smoke-free public housing (SFH) went into effect [1]. The new policy prohibits the use of tobacco in all indoor areas, including residential units and in outdoor areas within 25 feet of public housing and administrative office buildings. The rationale for the HUD decision is to protect residents from secondhand smoke (SHS) exposure, which leads to 41,000 deaths per year and is responsible for a wide range of serious health problems among adults and children [2,3,4].

Residents in public housing, a predominantly minority, low-income population, are at excess risk for SHS exposure at home when compared to the general public. This is largely due to higher smoking rates among residents living in public housing compared with the general population [5]. Additionally, most public housing residents live in multiunit housing (MUH) where SHS can transfer into smoke-free units from shared common areas in the building and nearby apartment units [6,7,8]. Thus, a smoke-free policy that prohibits smoking in all interior areas of the MUH buildings, including individual residencies, is the most effective way to eliminate SHS exposure.

The New York City Housing Authority (NYCHA) is the largest housing authority in the U.S., with more than 400,000 residents (average household income $24,336) [9,10]. A 2015 representative survey among NYCHA adult residents confirmed elevated smoking rates when compared to citywide averages (21% vs. 16%, *p* < 0.01) and that non-smoking NYCHA residents were more likely to smell cigarette smoke entering the home than nonsmoking New Yorkers overall (56% vs. 40%, *p* < 0.01) [11,12].

The purpose of this qualitative study was to explore NYCHA residents’ attitudes toward HUD’s new policy, and perceived barriers to policy implementation, and to elicit suggestions for optimizing policy implementation. The study was conducted in June and July 2017, one year before the new policy took into effect. The findings may help to inform local policy development and strategies for optimizing the implementation process.

## 2. Methods

### 2.1. Participants and Recruitment

We conducted 10 focus groups in June and July 2017 with 91 adult NYCHA residents living in Manhattan (7–12 participants for each group, mix of smokers and nonsmokers). Local community-based organizations (CBOs) that serve NYCHA residents assisted in recruitment. Study recruitment flyers were posted in NYCHA buildings, and the CBOs handed out flyers during resident meetings. The flyers provided a brief overview of the study. Interested residents who called the study telephone number on the flyer were screened for eligibility, and they were asked to sign up for a prescheduled focus group. Eligible residents lived in a NYCHA building for at least one year and were aged 18 years and older. The focus groups were conducted in multiple languages (four English, three Spanish, two Cantonese, and one bilingual Mandarin and Cantonese). Focus groups were conducted in five NYCHA developments (three in Baruch Houses, three in Smith Houses, one in Gomper Houses, one in Meltzer Houses, and two in Educational Alliance) located in Lower Manhattan.

The interview guide was informed by the socioecological model to explore multi-level factors that may impact policy implementation [13]. Specifically, the guide was developed to obtain data on compliance with the existing smoke-free policy, attitudes towards HUD’s new policy, and perceived barriers to and suggestions for increasing implementation effectiveness. Focus groups lasted approximately one hour and the participants received an honorarium of $30. The study protocol was approved by the Institutional Review Board of NYU School of Medicine. (s16-01965)

### 2.2. Data Analysis

All of the focus groups were audio recorded with the participants’ consent, and then transcribed verbatim and translated into English. Four members of the research team used an inductive (open coding) approach to identify broad analytic themes that are related to the study’s goals. Two investigators then engaged in an iterative process with the data to develop a codebook consisting of core codes and secondary codes related to themes. Coding differences were resolved through discussion with the larger research team and a reexamination of the transcripts. The codebook was then used for systematic coding of blocks of narrative text from the full transcripts. All of the interviews were coded and entered into a database utilizing the qualitative data analysis software ATLAS.ti 8.

## 3. Results

The 91 participants included 76 females and 15 males, 23 current smokers and 65 nonsmokers (three unknown). The main themes and subthemes are described below. Illustrative quotes are labelled by focus group number and language.

### 3.1. Concerns about Health Effects of SHS

Both smokers and nonsmokers were aware of the potential dangers of SHS exposure. But, it was nonsmokers who expressed significant concern about the negative health effects. Many shared specific examples of how they were regularly exposed to SHS in their apartments and the impact on their health and the health of their children. As one woman described: “My son···he has my asthma, and he get it because these people smoking. I have to put things in my house to prevent the smoke coming through my door” (#5 English).

### 3.2. Attitudes towards HUD’s New SFH Policy

Most of the participants supported the policy, citing the potential health benefits: “There will be better air and less negative side effects to health. It’s better for kids” (#6 Chinese). A few smokers acknowledged that the new policy could motivate smokers to quit: “It is a great idea honestly, because it will help us to quit smoking” (#4 Spanish). But some did not support the policy. For example, one smoker said: “you already can’t smoke in the building, so now you telling me you can’t smoke in the apartment, that you can’t go outside the building and smoke?? That don’t make sense to me” (#8 English).

### 3.3. Challenges to Policy Implementation and Enforcement

Both smokers and nonsmokers expressed concerns about a range of issues that they thought would create challenges to policy implementation.

#### 3.3.1. Violation of Current Smoke-Free Policy in the Building

At the time of this study, NYCHA had a partial smoke-free housing policy that prohibited smoking in indoor common areas and in management offices. Participants were generally aware of this policy and almost all described violations as a “pervasive” problem.

“They’re smoking in the staircase, in the hallway, in front of your door, even though it’s not their door... and the hallway is clouded full of smoke” (#8 English).

“It says ‘No Smoking’, but people stand in front of your door to smoke, and that smoke gets inside your apartment” (#3 Spanish).

One smoker acknowledged that he smoked in common areas because there were no consequences for violating the current policy: “I’ll go right out in the staircase. I bring my ashtray…Nobody tell me nothing” (#5 English). Despite frustration with this problem, residents were not always comfortable directly confronting smokers or reporting policy violations to NYCHA for several reasons, including lack of responsiveness to requests to stop and fear of retaliation. One resident described the consequences of complaining to NYCHA: “You know what he did? He slid a cigarette and a match under my door” (#3 Spanish).

Rather than try to confront smokers, participants described several strategies that they used to reduce SHS entering their homes. Some described putting towels under the door to block smoke from the hallways. One resident described putting plastic over her daughter’s window, “because my neighbors downstairs smoke. His room is below my daughters so smoke comes out of his window into hers” (#1 English).

#### 3.3.2. Enforcement

Enforcement was the most frequently mentioned challenge to policy implementation across all of the focus groups. Violations of the current smoke-free policy and other public housing policies created skepticism about NYCHA’s ability to enforce the expanded smoke-free policy, and smokers and nonsmokers had many questions about how NYCHA planned to operationalize enforcement (e.g., how they will detect and document violations).

“They got all these rules that they won’t enforce, right? Now, you just implementing another rule” (#8 English).

“Implementing is not easy...no one listens to you and no one enforce it” (#2 Chinese).

“I’m a smoker. How do they find out that you are smoking in your apartment if nobody complains?” (#1 English)

Residents also talked about the need for clearly designated areas for smokers to use tobacco. As one resident suggested: “… you have to create a common ground where smokers can go and smoke” (#8 English). Others thought that requiring smoking beyond 25 feet from the building might be unrealistic given the close proximity of public housing buildings in NYC. As one smoker noted: “These buildings are right next to each other. So you’re not 25 feet from your building but you’re 25 feet from somebody else’s building. So you can’t smoke nowhere near the project is what you’re saying” (#9 English).

#### 3.3.3. Marijuana Use in the Building

Our focus group questions did not specifically ask about marijuana use. Rather, the participants raised it in the context of discussing tobacco use violations. Most noted that marijuana use was as prevalent as cigarette use and similarly occurred in shared spaces (e.g., hallways and elevators) and individual units. Several complained that the smell was pervasive, one describing “choking” on a “cloud...so thick” (#8 English). For many, the failure of building management or residents to control marijuana smoke, even after complaints have been made, raised questions about the prospects for enforcement of the smoking policy.

#### 3.3.4. Fear of Eviction and Belief in Privacy Rights

Some participants expressed concern that the new smoke-free policy was simply another way to evict residents. As one smoker said: “I think they’re gonna try to put you out...There’s an agenda behind it…you can’t make somebody stop smoking cigarettes” (#9 English). Even nonsmokers were concerned about privacy rights: “I’m not a smoker or anything. This is your home, and you’re gonna come and tell me what to do in my own home?” (#1 English)

#### 3.3.5. Competing Priorities

Among participants in general, there was concern that the focus on implementing smoke-free policy would result in reduced attention to other priorities, including a need for building repairs, enforcing drug/marijuana laws, and improving sanitation and safety in the building. Several smokers resented being asked to change their behavior before many of these issues were addressed. For example, one resident urged NYCHA to: “Start fixing these apartments, then you can start making demands of that [no smoking in building]” (#5 English).

### 3.4. Recommendations for Policy Implementation

Participants, regardless of their attitudes toward the new policy, were eager to share their opinions about how to facilitate successful policy implementation.

#### 3.4.1. Educational Programs and Information Campaign

Almost all of the participants suggested a need to disseminate information to raise tenants’ awareness about the new policy (e.g., defining smoke-free areas, consequences of violations, etc.). Participants suggested that messages focus on the specific health benefits and the responsibility residents have to each other to create a safe and healthy environment. One participant suggested a message that urged residents “to be considerate of your neighbor” (#1 English).

#### 3.4.2. Smoking Cessation Services

Most participants believed that for the policy to be successfully implemented smokers would need help quitting, acknowledging that “it’s an addiction and it should be treated as such” (#9 English). Several recommended that NYCHA offer smoking cessation services to improve policy compliance. One participant (#5 English) suggested “giving free patches to people”.

#### 3.4.3. Resident Engagement

Many of the participants noted that it was very important for NYCHA to engage its tenants in policy planning and the implementation process. One resident expressed the view shared by others that, “They [NYCHA] have to speak with people…while there is no cooperation from the tenants, there won’t be any result” (#3 Spanish).

## 4. Discussion

This study provides important new insights into MUH residents’ concerns about the new SFH policy that will need to be addressed to optimize the implementation process. Among nonsmokers, support for the policy was driven primarily by the potential health benefits of reducing chronic exposure to SHS in their home. However, smokers and nonsmokers had similar concerns that were related to factors that may impede policy implementation. For example, a lack of attention to other building complaints, widespread violations of the current smoking policy, and the pervasiveness of illegal marijuana use, have created skepticism about resident compliance and eroded confidence that NYCHA has the capacity to enforce a new policy that would extend the smoking ban to include individual apartments.

Compliance and enforcement have been a challenge for public housing authorities (PHAs) that have implemented HUD-like policies [14]. For example, in 2012, the Boston Housing Authority (BHA) implemented a SFH policy that was similar to HUD’s. In a post implementation assessment, 51% of BHA respondents indicated that other residents “never” or “rarely” followed the new SFH policy, and 41% of respondents were dissatisfied with policy enforcement [15,16]. In a qualitative assessment of the BHA policy, a lack of outdoor smoking areas was cited by residents as one of the “greatest challenges to compliance” [16]. Smokers in this study were also concerned about how they could comply when the policy places such significant limits on where they can smoke outside. In dense urban settings, meeting the 25 feet requirement will be particularly challenging, but BHA’s experience supports the need to develop realistic alternatives for smokers if they are expected to move smoking outside.

Post implementation assessments of this policy also suggest that reluctance among residents and maintenance staff to report violations [16,17] may be at least one factor that has contributed to poor compliance and enforcement. We similarly found that, despite frustration with violations of the current policy, study participants were reluctant to directly confront smokers. Smoke-free laws in public spaces (e.g., restaurants) have generally relied on self-enforcement to be effective [18]. Likewise, the successful enforcement of SFH policy will depend on changes in social norms that create an environment where residents feel safe to promote smoke-free buildings through respectful engagement and that their efforts will be supported by the PHA and their community. To achieve this goal, strategies are needed to empower residents and staff by providing them with the knowledge and skills that they need to advocate for a healthy environment that includes smoke-free housing.

Residents expressed deep interest in engaging with NYCHA and had a wide range of suggestions on how to facilitate implementation. Early and ongoing engagement can provide opportunities for residents to generate ideas and participant in developing feasible community-driven plans to identify and report noncompliance that increase confidence that complaints will be acted upon, but does not increase the stigmatization of smokers.

Participant comments and data from BHA’s post implementation assessment indicate that cessation support for smokers will be an important part of any policy implementation plan. There are a number of free state and national cessation services. However, studies show that low income smokers are less likely to access cessation resources [19,20]. In NYC, NYCHA is partnering with the city health department and non-governmental organizations to develop a range of strategies to increase awareness of and access to evidence-based cessation services. This includes leveraging existing resources, like community health workers that are working with NYCHA residents and exploring new programs, like training residents as peer navigators to raise awareness about cessation services and help smokers to connect with these services [21].

Smoker and nonsmoker participants raised other concerns that were similar to those that were raised in other SFH policy assessments, including fears that the policy would be used to evict residents and privacy concerns. Our findings and previous research point to a need for clear communication that includes information about the rationale for the policy, including why it is necessary to include residential units and clear information about the consequences of violating the policy. We are aware of only one study that assessed messaging strategies that support or oppose smoking policies. Findings suggested that messages emphasizing the individual right to clean air and being a responsible neighbor might be effective in increasing resident acceptance of these policies [22]. Additional research is needed to inform communication and marketing campaigns that address the reasons residents may not support these policies and to foster social norms that support effective implementation of the new smoke-free policy.

Residents were also vocal about issues they believed were as important, or more important, than smoke-free policies, but they were not being addressed by the PHA. They were particularly frustrated by the widespread use of marijuana. It may not be clear to residents that NYCHA, like other PHAs, does not have authority to address marijuana use, as it is a police matter. The literature examining attitudes toward smoke-free policies in MUH has not described this phenomenon. Legalization of marijuana could help reduce this problem because residents could “take it outside” and not fear arrest. Until that happens, our findings suggest that to build community support for the HUD rule, additional strategies are needed to specifically address marijuana use in parallel to implementing the new smoke-free policy.

More generally, the new policy represents an opportunity for PHAs to take further steps to address tenants’ shared concerns about the complex array of issues that affect their health and living conditions by incorporating smoke-free housing policy into the larger healthy homes framework. This approach is being promoted by HUD, the Centers for Disease Control and Prevention, and other organizations nationally, and it is important to avoid the contradictions that arise when smoking is singled out and other violations that contribute to poor health are allowed [23,24].

Finally, our findings and other post implementation studies emphasize the need for ongoing monitoring and evaluation to assess trends in support and reasons for erosion of support, and to ensure that violations are minimized and the implementation and enforcement plans are effective [25]. With limited federal funding to support implementation and evaluation, PHAs should explore opportunities to partner with academic and community-based organizations to leverage resources and expertise to fill this gap.

The main limitation of this study is that the findings may not be generalizable to the larger population of NYCHA residents and other PHAs. Additionally, more females and non-smokers were enrolled in this study than males and smokers. The gender distribution in our sample largely reflected the full NYCHA population, which is predominately female. However, participants’ perceived barriers and concerns about the policy were similar to those that were described in prior studies of post implementation of HUD-like policies in other PHAs. A strength of this study is that it captured views across multiple race/ethnicity groups.

## 5. Conclusions

HUD’s new SFH policy promises to substantially reduce the health consequences of SHS exposure and motivate smoking cessation among public housing residents. Education campaigns, meaningful and ongoing community engagement with smokers and nonsmokers, increasing access to smoking cessation services, and addressing marijuana use and other health and safety issues are all important steps towards gaining community support for the policy and optimizing implementation.
